# Advances in the Identification of Quantitative Trait Loci and Genes Involved in Seed Vigor in Rice

**DOI:** 10.3389/fpls.2021.659307

**Published:** 2021-07-14

**Authors:** Jia Zhao, Yongqi He, Shuilai Huang, Zhoufei Wang

**Affiliations:** The Laboratory of Seed Science and Technology, Guangdong Key Laboratory of Plant Molecular Breeding, Guangdong Laboratory of Lingnan Modern Agriculture, State Key Laboratory for Conservation and Utilization of Subtropical Agro-Bioresources, South China Agricultural University, Guangzhou, China

**Keywords:** rice, seed development, seed dormancy, seed deterioration, seed germination

## Abstract

Seed vigor is a complex trait, including the seed germination, seedling emergence, and growth, as well as seed storability and stress tolerance, which is important for direct seeding in rice. Seed vigor is established during seed development, and its level is decreased during seed storage. Seed vigor is influenced by genetic and environmental factors during seed development, storage, and germination stages. A lot of factors, such as nutrient reserves, seed dying, seed dormancy, seed deterioration, stress conditions, and seed treatments, will influence seed vigor during seed development to germination stages. This review highlights the current advances on the identification of quantitative trait loci (QTLs) and regulatory genes involved in seed vigor at seed development, storage, and germination stages in rice. These identified QTLs and regulatory genes will contribute to the improvement of seed vigor by breeding, biotechnological, and treatment approaches.

## Introduction

Rice (*Oryza sativa* L.) is one of the most popular crops in the world. Direct seeding of rice at present is popular in many countries because of its low cost and convenience compared to the conventional transplantation (Farooq et al., 2011). Direct seeding can be classified into three types, including wet direct seeding, dry direct seeding, and water direct seeding, in rice (Mahender et al., 2015). However, the low seed germination, seedling establishment, and the poor seedling growth in field are the major constraints in the production of direct seeding. Seed vigor is an agronomical trait that determines the rapid and uniform germination, the final seedling emergence, and the seed storability and stress tolerance (Sun et al., 2007). Thus, rice varieties with high seed vigor are required for direct seeding. Seed vigor is regulated by a lot of genetic and environmental factors during seed development, storage, and germination stages. Here, the current advances on the identification of quantitative trait loci (QTLs) and regulatory genes involved in seed vigor at seed development, storage, and germination stages were mainly summarized in rice. Identification of the QTLs and genes involved in seed vigor will contribute to develop high-vigor varieties for direct seeding in rice.

## Characteristics of Seed Vigor

### Evaluation of Seed Vigor

Seed vigor is the sum of those properties that determine the germination activity and performance of seed lots in a wide range of environments (ISTA, 2015). Seed vigor is thus a complex trait, including the seed germination, seedling emergence and growth, and seed storability and stress tolerance (Sun et al., 2007; Finch-Savage and Bassel, 2016), in which the germination speed and uniformity are usually conducted using the indexes of germination potential, time to 50% germination (*T*_50_), and germination index in rice. The final germination percentage (GP) is used to test the seed viability, and the indexes of seedling percentage, vigor index, and seedling weight are conducted to evaluate seedling growth (Wang et al., 2010; He et al.,2019a,b). The above traits are usually used to evaluate seed vigor by traditional germination methods under normal, low-temperature, salinity, and flooding stress conditions (Wang et al.,2011a,b; Chamara et al., 2018). However, the conventional methods for seed vigor testing have several limitations as they involve visual assessment and are destructive. Thus, the non-contact- and non-destructive-type imaging techniques, such as visible imaging, hyperspectral imaging, and X-ray imaging, for conducting seed vigor might be an attractive alternative to the existing vigor assessment methods for rice (Mahajan et al., 2018; Zhang T. et al., 2020).

### Dynamic Changes of Seed Vigor

Seed vigor is influenced by a lot of factors, such as reserves accumulation and seed dormancy during development, seed deterioration during storage, and stress conditions and seed treatments during germination, in rice. Seed vigor progressively increases with physiological maturity (Bewley et al., 2013) and begins to decline before and after harvest (Finch-Savage and Bassel, 2016). Seed dormancy is established during seed maturation, which helps to prevent preharvest sprouting (PHS) at harvest stage (Sugimoto et al., 2010). However, deep dormancy will inhibit seed germination when sowing (Gubler et al., 2005). Thus, seed dormancy is established during development that has both advantages and disadvantages for seed vigor in terms of cultivation and utilization (Sugimoto et al., 2010). Seed vigor will ultimately decline during storage because of deterioration in rice (Sasaki et al., 2015; Finch-Savage and Bassel, 2016). Thus, seed longevity, the maintenance of viability during dry storage, is a crucial factor to preserve seed vigor (Pellizzaro et al., 2020). Additionally, seed vigor will be reduced by various stresses, such as salt, low temperature, and flooding stresses, during seed germination in rice (Wang et al.,2011a,b; Chamara et al., 2018), but it can be enhanced by seed priming treatment, an invigoration technique of low-vigor seeds (Rajjou et al., 2012). In most studies, the influencing factors of rice seed vigor is mainly considered at seed germination stage, whereas other stages are limitedly considered. To accurately characterize seed vigor of rice, the traits that influence seed vigor deserve to be studied systematically during seed development to germination stages. It will greatly help us in exploring the elite varieties for direct seeding, as well as unraveling its regulatory mechanisms and promoting variety breeding for high seed vigor in rice.

## Quantitative Trait Loci Involved in Seed Vigor

### Seed Development

Grain size is one of the key agronomic traits that determine grain yield in rice. Therefore, uncovering the genetic and molecular mechanisms of grain size is mainly conducted during seed development in rice, but seed vigor is less considered. More than 500 QTLs associated with grain size have been identified in rice, of which approximately 20 genes have been map-based cloned (Li et al., 2018). For example, *GS3* (Mao et al., 2010), *qGL3/GL3.1* (Qi et al., 2012; Zhang et al., 2012), *TGW6* (Ishimaru et al., 2013), *GLW7* (Si et al., 2016), *OsLG3* (Yu et al., 2017), *GL4* (Wu et al., 2017), *qLGY3/OsLG3b* (Liu et al., 2018; Yu et al., 2018a), *qTGW3/GL3.3* (Hu et al., 2018; Xia et al., 2018), and *GL6* (Wang et al., 2019) for gain length, and *GW2* (Song et al., 2007), *qSW5/GW5/GSE5* (Shomura et al., 2008; Weng et al., 2008; Duan et al., 2017), *GS5* (Li et al., 2011), *GW8* (Wang S. et al., 2012), and *TGW2* (Ruan et al., 2020) for gain width, as well as *GL7/GW7/SLG7* (Wang S. et al., 2015; Wang Y. et al., 2015; Zhou et al., 2015), *GW6a* (Song et al., 2015), and *GS9* (Zhao et al., 2018) for both gain length and width, have been cloned in rice. Several reports have shown that large seeds have the advantages of producing more vigorous seedlings (Douglas et al., 1994; Bockous and Shroyer, 1996), but the lack of a significant positive relation between seed weight and seedling dry weight has also been reported in several studies (Carleton and Cooper, 1972; Johnson and Wax, 1978). One rice population of recombinant inbred lines (RILs) was used to determine the genetic characteristics of the establishment of seed vigor at 4 (early), 5 (middle), and 6 weeks (late) after heading during seed development in 2 years in our previous study (Liu et al., 2014). We observed that the QTLs for seed vigor were rarely colocalized among the different maturity stages; more QTLs were expressed at the early maturity stage followed by the late and middle stages. The stably expressed QTLs for seed vigor are likely to coincide with QTL for grain size, low-temperature germinability, and seed dormancy (Liu et al., 2014). It is interesting to reveal that whether the above-described genes associated with grain size are also involved in controlling seed vigor in rice.

Seed dormancy is affected by multiple genes and environmental factors. Many QTLs have been identified for seed dormancy in rice. For example, two dormancy QTLs *qSdn-1* and *qSdn-5* have been obtained from the segregation analysis of the advanced backcross populations derived from the cross between cultivars N22 (strong dormancy) and Nanjing35 (weak dormancy) (Lu et al., 2011). Three putative QTLs, *Sdr6*, *Sdr9*, and *Sdr10*, for seed dormancy have been identified using chromosome segment substitution lines (CSSLs) derived from a cross between Nona Bokra (strong dormancy) and Koshihikari (weak dormancy) (Marzougui et al., 2012). A total of four, one, and three additive QTLs for seed dormancy have been identified at the early, middle, and late development stages, respectively, using one RIL population (Wang et al., 2014). A total of 12 and 27 QTLs for seed dormancy have been detected using CSSL and backcross inbred line (BIL) population, both derived from the same parents Nipponbare, a *japonica* cultivar with seed dormancy, and 9311, an *indica* cultivar lacking seed dormancy (Zhang C. et al., 2020). A genome-wide association study (GWAS) based on seed GP in freshly harvested seeds (FHSs) and after-ripened seeds (ARS) in 350 worldwide accessions has revealed that 16 loci significantly associated with GP in FHS and 38 in ARS (Magwa et al., 2016). Meanwhile, a GWAS has detected nine significant single-nucleotide polymorphisms (SNPs) associated with seed dormancy using an *indica*-only population consisting of 453 accessions, and a total of 212 candidate genes have been predicted (Lu et al., 2018). However, only few QTLs, such as *Sdr4* (Sugimoto et al., 2010) and *qSD1-2* (Ye et al., 2015), have been map-based cloned.

### Seed Longevity/Storability

Seed storability, defined as the ability to remain alive during storage, is an important agronomic and physiological characteristic. Several studies have detected some QTLs controlling seed storability in rice. For example, three putative QTLs for seed longevity, *qLG-2*, *qLG-4*, and *qLG-9*, have been detected using 98 BILs derived from a cross between a *japonica* variety Nipponbare and an *indica* variety Kasalath (Miura et al., 2002). A total of five and three QTLs associated with seed germination capability have been identified in various storage periods using two sets of RILs, derived from crosses between Milyang 23 and Tong 88-7 and between Dasanbyeo and TR22183, respectively (Jiang et al., 2011). A total of seven QTLs have been identified for seed storability using two segregating populations with N22 (*indica*) as a common parent, viz. a set of 122 BILs derived from the backcross Nanjing35 (*japonica*)/N22//Nanjing35 and another population comprising 189 RILs from the cross of USSR5 (*japonica*) and N22 (Lin et al., 2015). Seven putative QTLs have been identified for seed storability under natural storage using the BILs that was derived from a cross of a *japonica* cultivar, Nipponbare, and an *indica* cultivar, 9311 (Yuan et al., 2019). A GWAS has shown that eight major loci are associated with seed longevity parameters using 299 *indica* accessions, and the candidate genes conferring high seed longevity might be related to DNA repair and transcription, sugar metabolism, reactive oxygen species (ROS) scavenging, and embryonic/root development (Lee et al., 2019). These identified QTLs will be further studied to elucidate the mechanisms controlling seed longevity/storability; however, few QTLs, such as *qLG-9*, for seed longevity have been fine-mapped (Sasaki et al., 2015).

### Seed Germination

A total of 10 QTLs have been detected to control rice seed vigor, including germination rate, final GP, and germination index, using one RIL population derived from a cross between *japonica* Daguandao and *indica* IR28 under normal conditions (Wang et al., 2010). Most of the QTLs are likely to coincide with QTLs for seed weight, seed size, or seed dormancy, suggesting that the rice seed vigor might be correlated with seed weight, seed size, and seed dormancy (Wang et al., 2010). A total of 27 marker-trait associations with seed vigor have been detected using 540 rice cultivars (419 from China and 121 from Vietnam) under normal condition (Dang et al., 2014). Conditional QTL analysis identified that *qSV-1*, *qSV-5b*, *qSV-6a*, *qSV-6b*, and *qSV-11* influenced seedling establishment and that *qSV-5a*, *qSV-5c*, and *qSV-8* influenced only germination using an RIL population from a cross between *indica* cultivars ZS97 and MH63 (Xie et al., 2014). Recently, 43 QTLs have been identified governing the early germination and seedling vigor traits related to weed competitive ability in rice using a total of 167 BC_1_F_5_ selective introgression lines developed from a backcross population involving Weed Tolerant Rice-1 as the recipient parent and Y-134 as the donor parent (Dimaano et al., 2020).

Rice originated from tropical or subtropical areas is a low-temperature–sensitive crop. A large number of QTLs for seed germination have been detected under low-temperature conditions compared to that of normal conditions in rice. For example, four QTLs for low-temperature vigor of germination have been identified using F_2__:__3_ population, which included 200 individuals and lines derived from a cross of *indica* Milyang 23 and *japonica* Jileng 1 (Han et al., 2006). Three putative QTLs involved in low-temperature germination have been detected using an RIL population derived from a cross of *japonica* USSR5 and *indica* N22, and *qLTG-9* has been fine-mapped (Li et al., 2013). Using a mini core collection of 174 Chinese rice accessions, 22 QTLs for cold tolerance have been detected at the germination stage (Pan et al., 2015). A GWAS of low-temperature germinability in 187 rice natural accessions has revealed that 53 QTLs are associated with low-temperature germinability, and Stress-Associated Protein 16 (*OsSAP16*), coding for a zinc-finger domain protein, is a causal gene for one of the major QTLs (Wang X. et al., 2018). Recently, a total of 27 main-effect QTLs have been detected for germination and early seedling growth traits under low-temperature conditions using 230 introgression lines (ILs) in BC_1_F_7_ generation derived from the Weed Tolerant Rice-1 and Haoannong (Najeeb et al., 2020). A total of 20 QTLs for cold tolerance and cold recovery during germination have been identified in the subset of *japonica* accessions, whereas nine QTLs have been identified in the subset of *indica* accessions using a diversity panel of 257 rice accessions by GWAS approach (Thapa et al., 2020). Six and five QTLs for low-temperature tolerance have been detected during the germination and bud stages using RILs derived from *indica* rice H335 (tolerant) and *indica* rice CHA-1 (sensitive), and loci 3 has been detected during both the germination and bud stages (Yang et al., 2020). However, few QTLs, such as *qLTG3-1*, for low-temperature germinability have been cloned in rice (Fujino et al., 2008).

Salt tolerance of rice at the seed germination stage is one of the major determinants for the stable stand establishment in salinity soil. Several QTLs for seed germination have been detected under salt conditions in rice. For example, a total of 16 QTLs for seed imbibition and GP have been identified under control (water) and salt stress (100 mM NaCl) using one population of RILs, derived from a cross between a *japonica* rice landrace tolerant to salt stress and a sensitive *indica* rice variety (Wang et al., 2011b). A GWAS has revealed that 11 loci are associated with salt tolerance using 478 diverse rice accessions (Shi et al., 2017). A total of six QTLs for salt tolerance have been detected at the germination stage using 208 rice mini-core accessions by GWAS approach (Naveed et al., 2018). A total of 12 associated peaks have been detected for salt tolerance during seed germination by GWAS approach in rice, and the characterized 17 genes may contribute to salt tolerance (Yu et al., 2018b). Recently, a total of 13 QTLs have been identified for seed germination, i.e., 10 QTLs under H_2_O conditions and nine QTLs under salt conditions, using a BC_1_F_2_ population derived from the crossing Wujiaozhan (salt tolerant) and Nipponbare (salt sensitive), and LOC_Os06g10650, encoding tyrosine phosphatase family protein, might be the causal candidate gene for *qGR6.2* (Zeng et al., 2021).

A few QTLs for seed germination under flooding conditions have been detected in rice. For example, five QTLs for anaerobic germination have been detected on chromosomes 1 (*qAG-1-2*), 3 (*qAG-3-1*), 7 (*qAG-7-2*), and 9 (*qAG-9-1* and *qAG-9-2*) using a backcross population (Angaji et al., 2010). Using GWAS approach, 20 significant genes have been identified contributing to anaerobic germination in rice (Rohilla et al., 2020). Of them, two most relevant genes are *OsXDH1*, involved in purine catabolism pathway and acting as a scavenger of ROS in plants, and *SSXT*, which is a GRF1-interacting factor 3. Rapid elongation of the coleoptile is a perfect response to flooding during germination in rice. Nearly 20 SNP markers associated with the length of flooded coleoptiles have been detected using a pool of 432 *indica* varieties (Zhang et al., 2017). A total of 11 significant marker-trait associations for coleoptile length have been discovered by GWAS using 273 *japonica* accessions under submergence, and some candidate genes, such as auxin transporter *AUX1*, have been identified (Nghi et al., 2019). A novel QTL *qACE3.1* likely affecting the expression of genes involved in fermentative metabolism has been identified for coleoptile elongation under submergence using CSSLs (Nishimura et al., 2020). Recently, a total of 26 loci have been detected for coleoptile length and coleoptile diameter under submergence using 209 natural rice populations by GWAS approach, and four reliable candidate genes have been identified related to anaerobic germination (Su et al., 2021).

Seed vigor has not been selected as an important breeding trait in traditional breeding programs because of its quantitative inherence in rice. As described above, these detected QTLs for seed germination under various conditions in rice, and these candidate genes represent valuable resources for molecular breeding and genetic improvement of seed vigor during germination. However, only few QTLs have been map-based cloned. It is necessary to fine-map these regions and to find functional markers for marker-assisted selection in rice breeding programs for seed vigor. Usually, the *indica* rice presents stronger seed vigor during the germination stage than *japonica* rice under normal conditions (Wang et al., 2010). Typically, *indica* rice is more tolerant to salt stress than the *japonica* rice at seedling stage (Lee et al., 2003), whereas *japonica* rice exhibits better cold tolerance than *indica* rice (Zhang et al., 2005; Andaya and Tai, 2007; Cheng et al., 2007). However, this is not observed in salt and cold tolerance at the germination stage in rice (Wang et al.,2011a,b). It is interesting to reveal the mechanisms of seed vigor differences during seed germination between rice subspecies at various conditions.

## Genes Involved in Seed Vigor During Seed Development

### Nutrient Reserves

**FIGURE 1:**
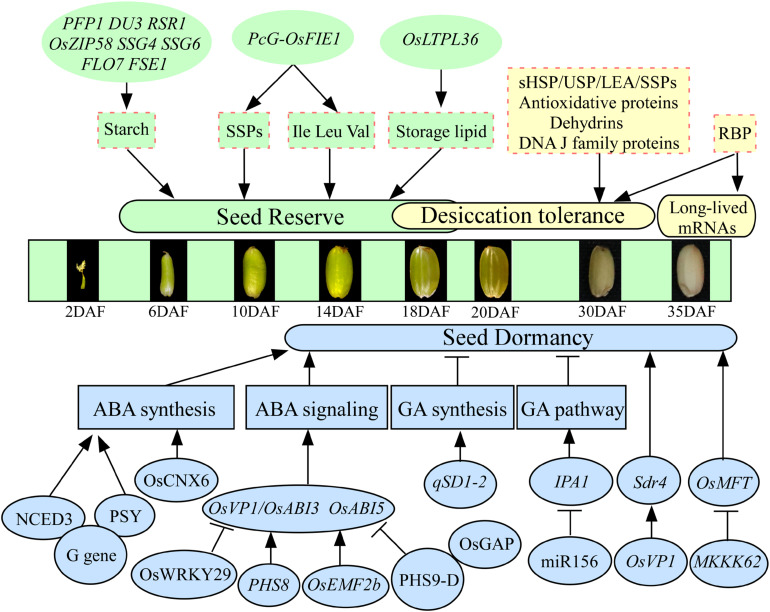
The regulatory genes involved in seed vigor during seed development in rice. The states of seed development according to Xu et al. (2008). Only several genes, such as *PFP1*, *PcG-OsFIE1*, and *OsLTPL36*, regulating reserves accumulation have been reported to be involved in seed vigor. The LEA, sHSP, USP, antioxidative proteins, dehydrins, and DNA J family are involved in the acquisition of desiccation tolerance. The RBP plays important roles in the stability of long-lived mRNAs. The genes regulate seed dormancy mainly through ABA and GA pathways in rice. The *G*, *PHS8*, *OsEMF2b*, *PHS9-D*, *OsCNX6*, and *OsWRKY29* regulate seed dormancy involved in ABA pathway, and *qSD1-2* and MIR156 regulate seed dormancy involved in GA pathway. Arrows and lines with slanted dashes indicate positive and negative effects, respectively. ABA, abscisic acid; GA, gibberellin; SSPs, seed storage proteins; LEA, late embryonic abundant; RBP, RNA-binding protein; *PFP1*, pyrophosphate-fructose 6-phosphate 1-phosphotransferase; *DU3*, mRNA cap-binding protein gene; *RSR1*, rice starch regulator1; *OsZIP58*, basic leucine zipper transcription factor; *SSG4*/*SSG6*, substandard starch grain; *FLO7*, floury endosperm; *FSE1*, floury shrunken endosperm 1; PcG, polycomb group; *OsFIE1*, fertilization-independent endosperm 1; *OsLTPL36*, lipid transporter; *Sdr4*, seed dormancy gene; *PHS8/9-D*, preharvest sprouting; *OsABI3/5*, ABA-responsive transcription factor; *OsVP1*, a homolog of the ABA-responsive transcription factor *OsABI3*; *OsGAP*, an interaction partner of the ABA receptor OsRCAR1; *OsEMF2b*, polycomb gene; G gene, stay-green G gene; *NCED3*, 9-*cis*-epoxycarotenoid dioxygenase; *PSY*, phytoene synthase; miR156, microRNA; *IPA1*, ideal plant architecture 1; *MKKK62*, protein kinases of MAPK cascade system; *OsMFT*, a homolog gene controlling wheat seed dormancy.

Most seeds contain large quantities of nutrient reserves, mainly carbohydrates, proteins, and lipids, biosynthesized and deposited during seed development. Only several genes regulating reserves accumulation have been reported to be involved in seed vigor in rice. For example, the mutation of rice *PFP1* (*Pyrophosphate-fructose 6-phosphate 1-phosphotransferase*) exhibits remarkably low grain weight and starch content but significantly increases protein and lipid content and then causes a significantly low germination rate (Figure 1; Chen et al., 2020). Imprinted polycomb group (PcG) complex binds to and alters chromatin condensation, resulting in the down-regulation of homeotic target gene expression. The PcG gene *Fertilization Independent Endosperm (FIE)* plays important roles in controlling seed development (Luo et al., 1999). The complex PcG-OsFIE1 of rice has been reported to regulate the development of embryo and endosperm and influences seed storage proteins and amino acids (Ile, Leu, and Val), which will affect the following seed germination (Huang et al., 2016). Downregulated expression of a lipid transporter *OsLTPL36* in plants will lead to decreasing seed setting rate and 1,000-grain weight, causing chalky endosperm, reducing fat acid content, and impeding seed germination in rice (Figure 1; Wang X. et al., 2015). These results indicate that the genes regulating nutrient reserves are vital components for seed vigor during seed development in rice. Starch is the most predominant storage substance, accounting for approximately 90% of the dry seed weight, in rice. A large number of regulatory genes involved in starch biosynthesis have been detected in rice. For example, the mRNA cap-binding protein gene *DU3* with pre-mRNA splicing, RNA nuclear export, and non-sense-mediated decay functions (Masayuki et al., 2008), rice starch regulator1 *RSR1* (Fu and Xue, 2010), basic leucine zipper transcription factor *OsZIP58* (Wang et al., 2013), substandard starch grain *SSG4* (Matsushima et al., 2014), *SSG6* (Matsushima et al., 2016), floury endosperm *FLO7* (Zhang et al., 2016), and floury shrunken endosperm1 *FSE1* (Long et al., 2018) have been demonstrated to affect starch biosynthesis and endosperm development in rice (Figure 1). However, whether these genes are involved in the regulation of seed vigor is still unclear in rice. Additionally, the relationships between the seed storage proteins, including glutelins, prolamins, and globulin, with seed vigor are understood limitedly in rice, although several genes involved in the accumulation of storage proteins have been reported (Lee et al., 2015).

### Desiccation Tolerance

Desiccation tolerance ensures seed survival through natural maturation or artificial drying, which is important for the establishment of seed vigor. Rice seeds are orthodox seeds, which tolerate water losses of up to 7% of their water content and can be stored at low temperature. In rice, dehydration begins 10–20 days after flowering (DAF), and desiccation phase occurs in the period from 20 to 40 DAF (Figure 1; Sano et al., 2013a). Proteomic analysis has revealed that late embryogenesis abundant (LEA) proteins, small heat shock proteins (sHSPs), universal stress proteins (USPs), antioxidative proteins, dehydrins, and DNA J family accumulate at the beginning of dehydration and remain at a high level in the desiccation phase, suggesting that these proteins are involved in the acquisition of desiccation tolerance in rice (Sano et al., 2013a; He and Yang, 2013; Wang W. Q. et al., 2015). Interestingly, it has been reported that more than 17,000 different mRNA species are stored in mature dry seeds of rice (Howell et al., 2009), which are designated “long-lived mRNAs” because they remain active and translatable even if seeds undergo severe desiccation. Why can these mRNAs live long after seed desiccation or artificially drying? Previous study has shown that the RNA-binding protein (RBP) may have important roles in the stability of long-lived mRNAs in rice (Figure 1; Sano et al., 2013b). After seed imbibition, a population of stored mRNAs is selectively loaded into polysomes, and the mRNAs, involved in processes such as redox, glycolysis, and protein synthesis, are actively translated for germination (Sano et al., 2020). Additionally, the selective oxidation of stored mRNAs has been reported to be involved in dormancy release during after-ripening, and non-selective oxidation and degradation of stored mRNAs have been reported to be involved in seed deterioration during long-term storage (Sano et al., 2012, 2020). However, which specific long-lived mRNAs are sufficient for the seed germination, dormancy, and storability is still limitedly understood.

### Seed Dormancy

Seed dormancy has both advantages and disadvantages for seed vigor. In rice, most of the dormancy-related genes are involved in the synthesis or signal transduction of gibberellin (GA) and abscisic acid (ABA; Figure 1). For example, the classical stay-green *G* gene has conserved functions in controlling seed dormancy in soybean, rice, and *Arabidopsis*. Stay green *G* gene affects seed dormancy via the interactions with 9-*cis*-epoxycarotenoid dioxygenase NCED3 and phytoene synthase PSY and in turn modulates ABA synthesis (Wang M. et al., 2018). The expression of the first rice seed dormancy gene *Seed dormancy 4* (*Sdr4*), a key gene controlling PHS, is controlled by the ABA-responsive transcription factor *OsVP1* (Sugimoto et al., 2010). A rice mutant, *phs8*, encodes a starch debranching enzyme named isoamylase1, and it exhibits PHS phenotype by affecting ABA signaling. Mutation in *PHS8* results in the decreased expression of *OsABI3* and *OsABI5*, as well as reduced sensitivity to ABA (Du et al., 2018). Polycomb gene *OsEMF2b* could directly bind to the promoter of *OsVP1* and could affect H3K27me3 enrichments of *OsVP1* in seedling, which will influence seed dormancy (Chen et al., 2017). Rice PHS9-D could interact with OsGAP, a partner of ABA receptor OsRCAR1, and *PHS9* and *OsGAP* play important roles in the regulation of rice PHS through the integration of ROS and ABA signaling (Xu et al., 2019). Additionally, mutation of *qSD1-2*, which controls GA synthesis, leads to decreasing GA levels and enhancing seed dormancy (Ye et al., 2015). The grain yield modulator miR156 regulates seed dormancy involved in GA pathway through de-repression of the miR156 target gene *ideal plant architecture 1* (*IPA1*; Miao et al., 2019). The transcription factor *IPA1* reduces unproductive tillers and increases grains per panicle, which results in improved rice yield (Jiao et al., 2010; Miura et al., 2010; Lu et al., 2013). Protein kinase *MKKK62* has been reported to negatively control seed dormancy in rice, and it regulates seed dormancy through altering *OsMFT* transcription (Figure 1; Mao et al., 2019). Molybdenum cofactor (MoCo) is required for ABA biosynthesis in plants. Recently, map-based cloning has shown that *OsCNX6* encoding homologs of MoaE participate in MoCo biosynthesis and is essential for rice development, especially for seed dormancy and germination (Liu et al., 2019). A new ABA signaling repressor *OsWRKY29* represses seed dormancy by directly down-regulating the expression of *OsABF1* and *OsVP1* (Zhou et al., 2020). As mentioned previously, the genes regulate seed dormancy mainly through ABA and GA pathways in rice. However, the regulations of other hormones (ethylene, brassinosteroids, salicylic acid, cytokinin, auxin, and jasmonic acid) and radicals (reactive oxygen and nitrogen species), as well as their crosstalk networks, on seed dormancy need to be further investigated. Additionally, proteomic studies showed that dormancy release is involved in metabolism, energy production, protein synthesis and destination, storage protein, cell growth and division, signal transduction, cell defense, and rescue in rice (Xu et al., 2016). These identified genes need to be further confirmed. Because of the selection for the rapid and uniform germination during rice domestication and breeding activities, many modern cultivars have lost part or all of their dormancy at development stage (Gu et al., 2008). Therefore, seeds of rice require a well-balanced level of seed dormancy; data on how to use the above genes to develop the cultivars with deep dormancy during development stage but non-deep dormancy when seed sowing are urgent (Cheng et al., 2014).

## Genes Involved in Seed Vigor During Seed Storage

### Lipid Peroxidation

**FIGURE 2:**
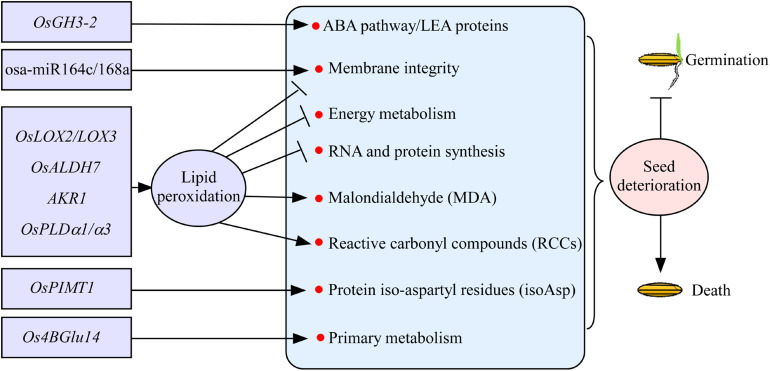
The regulatory genes involved in seed deterioration during seed storage in rice. Most of the genes, such as *OsLOX2/LOX3*, *OsALDH7*, *AKR1*, and *OsPLD*α*1/*α*3*, regulate seed deterioration mainly through lipid peroxidation. Rice *OsPIMT1*, osa-miR164c/168a, *Os4BGlu14*, and *OsGH3-2* regulate seed deterioration involved in the information of protein iso-aspartyl residues, membrane integrity, primary metabolism, and ABA pathway, respectively. Arrows and lines with slanted dashes indicate positive and negative effects, respectively. osa-miR164c/168a, microRNAs; *OsLOX2/LOX3*, lipoxygenase 2/3; *OsALDH7*, aldehyde dehydrogenases; *AKR1*, aldo-ketoreductase; *OsPLD*α*1/*α*3*, phospholipase D; *OsPIMT1*, protein–lisoaspartyl methyltransferase; *Os4BGlu14*, a monolignol β-glucosidase; *OsGH3-2*, indole-3-acetic acid (IAA)–amido synthetase gene *GRETCHEN HAGEN3-2*.

Seed deterioration occurs always during seed storage in rice, resulting in the gradually loss of seed vigor. Seed deterioration has shown to be regulated by a number of genetic factors in rice. Of them, lipid peroxidation is a major factor influencing seed vigor (Figure 2). Lipid peroxidation is catalyzed by the enzyme lipoxygenase 3 (LOX3), and the suppression of *LOX3* expression increases grain storability in rice (Xu et al., 2015). Similarly, *OsLOX2* acts in the opposite directions between seed germination and seed longevity. Appropriate repression of the *OsLOX2* gene may delay the aging process during seed storage (Huang et al., 2014). Aldehyde dehydrogenases (ALDHs) catalyze the irreversible oxidation of a wide range of reactive aldehydes to their corresponding carboxylic acids. Rice *OsALDH7* plays an important role in maintaining seed viability by detoxifying the aldehydes generated with lipid peroxidation (Shin et al., 2009). Lipid peroxidation mediates the reactive carbonyl compounds (RCCs) and non-enzymatic modifications of proteins through Maillard and Amadori products to reduce seed vigor. Rice aldo-ketoreductase (AKR1) enzyme detoxifies cytotoxic compounds, which results in lower levels of cytotoxic compounds and glycation products, to improve seed vigor (Nisarga et al., 2017). Lipid degradation caused by phospholipase D (PLD) activity is known to be responsible for seed deterioration (Wang S. et al., 2012). The transcript levels of *OsPLD*α*1/*α*3* will be increased during seed aging in rice, indicating that *OsPLD* plays an important role in seed deterioration (Figure 2; Nisarga et al., 2017). Overall, the lipid peroxidation causes the loss of membrane integrity, reduced energy metabolism, impairment of RNA and protein synthesis, and formation of malondialdehyde and RCCs, which will lead to seed deterioration during storage in rice (Figure 2; Kapoor et al., 2011).

### Other Factors

Damaged proteins containing abnormal isoaspartyl (isoAsp) accumulate as seeds age, and the abnormality is thought to undermine seed vigor. Protein–lisoaspartyl methyltransferase (PIMT) involved in both seed longevity and germination vigor is first reported in *Arabidopsis* (Ogé et al., 2008). In rice, the improvement of seed longevity by *OsPIMT1* is probably due to the repair of detrimental isoAsp containing proteins (Figure 2; Wei et al., 2015). MicroRNAs (miRNAs) are the key regulators of gene expression in many important biological processes of plants. Recently, miRNAs osa-miR164c and osa-miR168a have been shown to regulate seed vigor under aging condition by influencing the membrane integrity of seeds in rice (Figure 2; Zhou et al., 2020). Rice *Os4BGlu14*, a monolignol β-glucosidase, plays a negative role in seed longevity by affecting primary metabolism during seed development and aging (Ren et al., 2020). Recently, indole-3-acetic acid (IAA)–amido synthetase gene *GRETCHEN HAGEN3-2* (*OsGH3-2*) has been detected controlling seed storability, and it acts as a negative regulator of seed storability by modulating many genes related to the ABA pathway and probably subsequently LEA proteins at the transcription level (Yuan et al., 2021). Proteomic study has shown that several redox regulation proteins, mainly glutathione-related proteins, and some disease/defense proteins, including DNA-damage-repair/toleration proteins, might be correlated with seed storability (Gao et al., 2016). Metabolomic study has shown that raffinose has potential roles in seed storability (Yan et al., 2018). Altogether, only few factors involved in seed deterioration have been verified in rice (Figure 2). The underlying genetic basis of seed vigor during seed storage needs to be further studied in rice.

## Genes Involved in Seed Vigor During Seed Germination

### Seed Reserve Utilization

**FIGURE 3:**
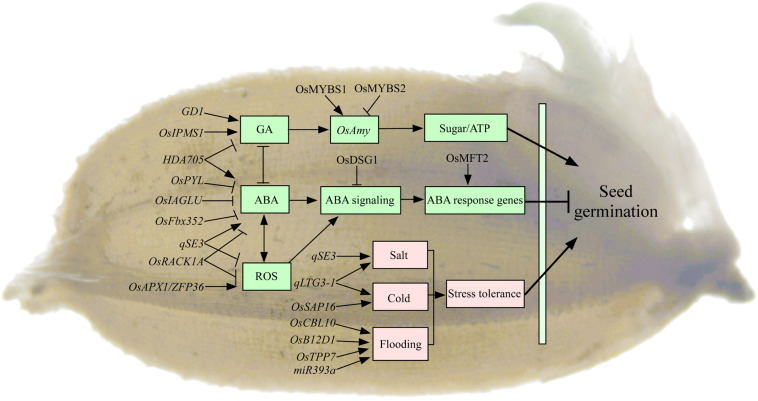
The regulatory genes involved in seed germination in rice. Most of the genes regulate seed germination mainly through GA, ABA, and ROS pathways in rice. Rice *GD1*, *OsIPMS1*, and *MYBS1/MYBS2* regulate seed germination by influencing α*Amy* expression through GA pathway. Rice *OsPYL*, *OsDSG1*, *OsIAGLU*, *OsFbx352*, and *OsMFT2* regulate seed germination involved in ABA pathway. Rice *HDA705* regulates seed germination through GA and ABA pathways. Rice *qSE3*, *OsAPX1*, and *OsRACK1A* regulate seed germination involved in the crosstalk between ABA and ROS pathways. A number of genes have been reported to be involved in seed germination under stress conditions. Rice *qLTG3-1* and *OsSAP16* regulate low-temperature germinability, and *qSE3* regulates seed germination under salt condition, and *OsB12D1*, *TPP7*, miR393a, and *OsCBL10* regulate seed germination under flooding condition. Arrows and lines with slanted dashes indicate positive and negative effects, respectively. ABA, abscisic acid; GA, gibberellin; ROS, reactive oxygen species; ATP, adenosine-triphosphate; LTG, low-temperature germinability; *GD1*, germination-defective 1; *OsIPMS1*, isopropylmalate synthase; *OsAmy*, alpha-amylases; *HDA705*, RPD3/HDA1-type histone deacetylase; *OsFbx352*, F-box protein; *OsPYL*, pyrabactin resistance 1 like; *OsDSG1*, delayed seed germination 1; *OsMFT2*, *mother of FT and TFL 1*; *OsIAGLU*, indole-3-acetate betaglucosyltransferase; *qSE3*, QTL for seedling establishment under salinity stress; *OsRACK1A*, rice receptor for activated C kinase 1; *OsAPX1*, ascorbate peroxidase; *ZFP36*, zinc finger transcription factor; *qLTG3-1*, QTL for seed germination under low temperature; *OsSAP16*, stress-associated protein 16; *OsB12D1*, an unknown subgroup protein of the barley aleurone and embryo; miR393a, MicroRNAs; *TPP7*, *trehalose 6 phosphate phosphatase 7*; *OsCBL10*, calcineurin B–like protein 10.

Seed germination begins with the imbibition of water and ends with the protrusion of the coleoptile and/or radicle (Bewley, 1997). The mobilization of stored starch upon imbibition is important to supply the energy and nutrients for seed germination and seedling growth. In rice, most α-amylases (*RAmy1*, *RAmy2*, and *RAmy3*) are exclusively expressed in the germinating seeds, which are critical to motivate the stored starch to nourish the developing seedling (Figure 3; Damaris et al., 2019). The expression of α*Amy* genes is negatively regulated by sugar in embryo during seed germination in rice (Chen et al., 2019). The on/off switch of α*Amy* expression is regulated by two MYB transcription factors competing for the same promoter element. MYBS1 promotes α*Amy* expression under sugar starvation, whereas MYBS2 represses it (Chen et al., 2019). Meanwhile, α*Amy* expression is positively regulated by GA in endosperm (Damaris et al., 2019). However, few studies have reported on the roles of storage lipid mobilization for seed germination in rice. Recently, the lipidome analysis has revealed the distinct patterns of molecular species distribution in individual lipid classes and displayed the metabolic connections between lipid mobilization and rice seedling growth (Dolui and Vijayaraj, 2020). The roles of lipid homeostasis for seed germination are still limitedly understood in rice. Seedling establishment begins with the appearance of the radicle and terminates when the seedling has exhausted the seed’s energy reserves and starts to carry out photosynthesis. The trait of seed reserve utilization, e.g., the conversion efficiency of utilized seed reserve into seedling tissue, plays important roles in rice vigorous seedling growth (Cheng et al., 2013). However, only three α*Amy* genes (*OsAmy3B*, *OsAmy3C*, and *OsAmy3E*) and sucrose synthase (*OsSus2*, *OsSus3*, and *OsSus4*) have been reported to be involved in seed reserve utilization in rice (Cheng et al., 2015). The genetic characteristics of seed reserve utilization are still unclear in rice.

### Signal Pathway

It has been reported that several genes regulate seed germination involved in GA pathway in rice (Figure 3). For example, rice *GD1* (*germination-defective1*) regulates seed germination through directly or indirectly regulating GA_4_ level and carbohydrate homeostasis (Guo et al., 2013). Disruption of isopropylmalate synthase *OsIPMS1* results in low seed vigor, which might be tightly associated with the reduction of amino acids and GA biosynthesis, as well as energy level, in germinating seeds (He et al., 2019a). Hormone ABA is an essential repressor of seed germination (Figure 3). Transgenic rice plants expressing *OsPYL/RCAR5* (*pyrabactin resistance 1 like/regulatory components of ABA receptors*) are hypersensitive to ABA during seed germination and early seedling growth (Kim et al., 2012). Rice Delayed Seed Germination 1 (OsDSG1) could bind to OsABI3 that is a major regulator of ABA signaling in germinating seeds (Park et al., 2010). Rice F-box protein *OsFbx352* plays a regulatory role in the glucose-induced suppression of seed germination by targeting ABA metabolism, including ABA synthesis (*OsNCED2* and *OsNCED3*) and ABA catabolism (*OsABA-ox2* and *OsABA-ox3*), in the presence of glucose (Song et al., 2012). Rice *mother of FT and TFL 1* (*OsMFT2*) negatively regulates seed germination, and it positively regulates ABA response genes through interacting with three bZIP transcription factors *OsbZIP23/66/72* (Song et al., 2020). Several genes regulate seed vigor involved in the crosstalk between hormone and/or ROS pathways in rice (Figure 3). For example, the overexpression of rice *HDA705*, a RPD3/HDA1-type *histone deacetylase* (*HDAC*) gene, in plants delays seed germination because of the down-regulated expression of GA biosynthetic genes and up-regulation of ABA biosynthetic genes (Zhao et al., 2016). Disruption of indole-3-acetate betaglucosyltransferase *OsIAGLU* gene results in low seed vigor in rice. The regulation of seed vigor by *OsIAGLU* is through modulating auxin (IAA) and ABA levels to alert *OsABIs* expression in germinating seeds of rice (He et al., 2020b). Rice *OsAPX1* coding an ascorbate peroxidase (APX) has the most affinity for H_2_O_2_ (substrate; a type of ROS) that is a direct target of zinc finger transcription factor *ZFP36*. The LEA protein OsLEA5 interacts with ZFP36 to coregulate OsAPX1 in seed germination in rice (Huang et al., 2018). Rice receptor for activated C kinase 1 (*OsRACK1A*), one member of the most important WD repeat–containing family of proteins, positively regulates seed germination by controlling endogenous levels of ABA and H_2_O_2_ and their interactions (Zhang et al., 2014). RNA sequencing approach has been used to understand the signaling responses in the initial imbibition (8 h) of rice seed germination. The majority of signaling response-related genes are hormone- and transcription factor–related genes, and the largest number of genes belongs to the AP2-domain–containing regulators (He et al., 2020a). Similar with above seed dormancy, the genes regulate seed germination mainly through ABA and GA pathways in rice. However, the regulations of other hormones, radicals, and transcription factors, as well as their crosstalk networks, on seed germination also need to be further investigated.

### Stress Tolerance

Seed germination of rice is also influenced by a series of abiotic factors, such as low temperature, salt, and flooding stress. A number of genes have been reported to be involved in rice seed germination under stress conditions (Figure 3). For example, the low-temperature germinability *qLTG3-1* is preferentially expressed in the coleorhiza and epiblast of rice, and it increases seed germination through the cell vacuolation and tissue relaxation (Fujino et al., 2004, 2008). *Stress-Associated Protein* 16 (*OsSAP16*), coding for a zinc-finger domain protein, is a causal gene for the major low-temperature germinability QTL in rice. The variation in expression of the *OsSAP16* gene contributes to low-temperature germinability variation in rice (Wang X. et al., 2018). One major QTL *qSE3*, which encodes a K^+^ transporter gene *OsHAK21*, positively regulates seed germination and seedling establishment by increasing ABA biosynthesis, activating ABA signaling responses, and decreasing H_2_O_2_ level in germinating seeds under salinity stress in rice (He et al., 2019b). Rice *OsB12D1* belongs to a function unknown subgroup of the *Balem* (*Barley aleurone and embryo*) protein that positively regulates flooding tolerance during seed germination and early seedling growth. This tolerance regulated by *OsB12D1* is possibly through enhancing electron transport to mediate Fe and oxygen availability under flooded conditions (He et al., 2014). The *trehalose 6 phosphate phosphatase 7* (*TPP7*) gene plays a key role in the mobilization of starch under water in terms of coleoptile elongation and embryo development (Kretzschmar et al., 2015). The miR393a/target plays a role in coleoptile elongation and stomata development via the modulation of auxin signaling during seed germination and seedling establishment under submergence (Guo et al., 2016). Natural variation in the promoter of rice *Calcineurin B–like Protein10* (*OsCBL10*) has been identified affecting flooding tolerance during seed germination among subspecies (Ye et al., 2018). The adaptation to flooding stress during rice germination is associated with two different *OsCBL10* promoters, which in turn affects *OsCBL10* expression in different cultivars and negatively affects calcineurin B–like protein-interacting protein kinase (OsCIPK15) protein accumulation and its downstream cascade (Ye et al., 2018). Altogether, these above identified genes might be promising genes to enhance seedling establishment for direct seeding under various field conditions in rice. Seed germination is a complex trait that is influenced by many genetic and environmental factors, but the key event(s) associated with seed germination are still poorly understood. It is necessary to integrate the available genomic, transcriptomic, and metabolomic data in order to comprehensively understand the process of germination in rice (He and Yang, 2013; Yang et al., 2019).

### Seed Priming

Seed priming techniques limit the availability of water to the seed, in which there is sufficient water to initiate germination processes while preventing radicle emergence (Heydecker et al., 1973; Bewley et al., 2013; Cheng et al., 2017). Priming is widely used to enhance seed vigor in terms of the traits of germination potential and stress tolerance (Sheteiwy et al., 2016; Singh et al., 2020). On-farm seed priming with water in maize, rice, and chickpea in India has been chosen as a low-cost, low-risk intervention appropriate to the farmers’ needs (Harris et al., 1999). The activation of DNA repair and antioxidant mechanisms, the *de novo* synthesis of nucleic acids and proteins, and the production of ATP when seed rehydration are all generally agreed benefits of seed priming for improving seed vigor (Balestrazzi et al., 2010; Paparella et al., 2015). However, few genes have been reported to be involved in seed priming to date in rice. Proteomics have identified that a series of proteins, such as those related to metabolism, storage, protein synthesis, ROS scavenging, and signaling, as well as stress response, might be involved in seed priming in rice (He and Yang, 2013). Recently, our data have demonstrated that the expression of *OsIPMS1* contributes to seed priming due to ATP production (He et al., 2019a). Seed priming involves a dehydration step after the controlled imbibition back to the original moisture content of the treated seeds. Thus, the choice of correct stop time-point for seed priming is a critical step, but it is still difficult to monitor in rice because of the variation of priming effects among varieties and seed batches. The phenotypic, physiological, and biochemical markers are difficultly used in the control of priming process because these traits are affected by environmental factors (Cheng et al., 2017). To unravel molecular markers of seed priming in rice, it is possible to characterize the genes as ideal candidate biomarkers for rice seed priming (Cheng et al., 2017; He et al., 2019a). Seed priming with various priming agents has been reported to improve seed vigor under different stress conditions such as salinity, cold, and drought in rice (Salah et al., 2015; Hussain et al., 2016; Samota et al., 2017; Du et al., 2019; Subramanyam et al., 2019). However, the mechanisms of seed priming contributing to seed vigor under stress conditions are limitedly understood in rice. The molecular markers to determine the best stop time-point of priming also need to be investigated further in the future.

## Conclusion

In this review, we have summarized findings about the QTLs and genes associated with seed vigor in rice. Seed vigor is a complex trait that is determined by a lot of factors during seed development, storage, and germination stages in rice. Further developing a systems approach to accurately evaluate seed vigor will greatly help in unraveling its molecular mechanisms. Nowadays, the studies of seed vigor are mainly focused on seed dormancy and germination, whereas there are few studies on seed longevity and seed priming associated with seed vigor in rice. Seed vigor is regulated by a very complicated network of signaling and gene expression in rice, and its molecular mechanisms are still largely unknown. Postgenomics studies at the three levels of transcripts, proteins, and metabolites have provided important new information about the mechanisms underlying seed vigor in rice. However, only limited factors have been verified. Characterizing the more seed vigor-related genes will provide us a better understanding of the regulatory mechanisms of seed vigor in rice, which may allow us to enhance seed vigor by breeding and seed treatment approaches.

## Author Contributions

ZW designed the manuscript. ZW, YH, JZ, and SH wrote the manuscript. All authors contributed to the article and approved the submitted version.

## Conflict of Interest

The authors declare that the research was conducted in the absence of any commercial or financial relationships that could be construed as a potential conflict of interest.
